# Treatment of forefoot problems in older people: study protocol for a randomised clinical trial comparing podiatric treatment to standardised shoe advice

**DOI:** 10.1186/1757-1146-4-11

**Published:** 2011-03-31

**Authors:** Babette C van der Zwaard, Petra JM Elders, Dirk L Knol, Kees J Gorter, Louis Peeraer, Daniëlle AWM van der Windt, Henriëtte E van der Horst

**Affiliations:** 1 EMGO Institute, VU University Medical Centre, Amsterdam, The Netherlands; 2 Podiatry department, Fontys University for Applied Sciences, Eindhoven, The Netherlands; 3 Department of Epidemiology and Biostatistics, VU University Medical Centre, Amsterdam, The Netherlands; 4 Department of General Practice, University Medical Centre Utrecht, Utrecht, the Netherlands; 5 Arthritis Research National Primary Care Centre, Primary Care Sciences, Keele University, UK

## Abstract

**Background:**

Foot problems in general and forefoot problems in particular can lead to a decrease in mobility and a higher risk of falling. Forefoot problems increase with age and are more common in women than in men. Around 20% of people over 65 suffer from non-traumatic foot problems and 60% of these problems are localised in the forefoot. Little is known about the best way to treat forefoot problems in older people. The aim of this study is to compare the effects of two common modes of treatment in the Netherlands: shoe advice and podiatric treatment. This paper describes the design of this study.

**Methods:**

The study is designed as a pragmatic randomised clinical trial (RCT) with 2 parallel intervention groups. People aged 50 years and over who have visited their general practitioner (GP) with non traumatic pain in the forefoot in the preceding year and those who will visit their GP during the recruitment period with a similar complaint will be recruited for this study. Participants must be able to walk unaided for 7 metres and be able to fill in questionnaires. Exclusion criteria are: rheumatoid arthritis, neuropathy of the foot or pain caused by skin problems (e.g. warts, eczema). Inclusion and exclusion criteria will be assessed by a screening questionnaire and baseline assessment. Those consenting to participation will be randomly assigned to either a group receiving a standardised shoe advice leaflet (n = 100) or a group receiving podiatric treatment (n = 100). Primary outcomes will be the severity of forefoot pain (0-10 on a numerical rating scale) and foot function (Foot Function 5-pts Index and Manchester Foot Pain and Disability Index). Treatment adherence, social participation and quality of life will be the secondary outcomes. All outcomes will be obtained through self-administered questionnaires at the start of the study and after 3, 6, 9 and 12 months. Data will be analysed according to the "intention-to-treat" principle using multilevel level analysis.

**Discussion:**

Strength of this study is the comparison between two common primary care treatments for forefoot problems, ensuring a high external validity of this trial.

**Trial registration:**

Netherlands Trial Register (NTR): NTR2212

## Background

Pain and discomfort due to foot problems are common and increase with age. Population-based surveys have estimated the prevalence of foot problems between 14.9 and 41.9% for people aged 50 years and older [1-4]. The prevalence of foot problems was found to be higher in women than in men [2,5]. Not all foot problems or foot deformities lead to pain or functional limitations [5-7] but people with disabling foot pain have been shown to experience a lower degree of well-being [2] and to have a higher risk of a decrease in mobility [2,8] and falling [9,10].

Forefoot problems including metatarsalgia, hallux valgus and hallux rigidus are the most common foot problems in older people [2,5,9]. A community-based study among 5689 older people in the Dutch area of Apeldoorn (a mixed urban-rural area) showed a prevalence rate of forefoot problems of 60% within the group of people reporting non-traumatic foot problems (n = 1130) [2]. In an English population survey among 3417 adults, more than one-third indicated to have pain in the great toe or in the first metatarsophalangeal joint (MTP-joint), and 9.5% indicated to be disabled by their foot problem [5].

Underreporting of foot problems is an acknowledged phenomenon in healthcare [9-11]. The community-based survey among older people in The Netherlands [9] showed that only 56% of the respondents sought health care, by consulting their general practitioner (GP) (46%), a medical specialist (36%) (mainly orthopaedic surgeons) or an allied health care professional (18%) (mainly podiatrists). A GP will commonly refer non-traumatic foot problems to a podiatrist or will treat the patient him or herself. Dutch podiatric care starts with an assessment which includes medical history, detailed analysis of the anatomical relationships within the foot and both a postural and a gait analysis [12]. Treatment may consist of (or a combination of) construction of full length podiatric insoles, silicone toe devices, (shoe) advice or basic foot and nail care. The treatment by the GP usually consists of prescribing pain medication (simple analgesic or non-steroidal inflammatory drugs [NSAIDs]) or by giving lifestyle advice (e.g. try to lose weight, improve shoe wear) [13].

The primary cause of non-traumatic forefoot problems can be very diverse and includes the possible influence of ill-fitting shoes [14,15]. Some evidence exists that ill-fitting footwear is associated with foot problems such as corns, calluses, hallux valgus and lesser toe deformities. Both the advice to buy well-fitting shoes and referral to a podiatrist are common treatment modalities of Dutch GPs. Whether these treatments actually lead to improvement of non-traumatic forefoot problems is not known, nor is it known if one treatment is more effective than the other.

The aim of this article is to describe the protocol of a pragmatic randomised clinical trial to compare the effectiveness of two common treatments for forefoot problems in older people and to discuss (or comment on) the choices made in the design. The primary objective of the trial is to investigate the effectiveness of podiatric treatment versus standardised shoe advice in people aged 50 years and older with disabling forefoot pain. The secondary objective is to conduct a process evaluation of the podiatric treatment provided for forefoot pain in this trial.

## Method

### Trial design

**Figure 1:**
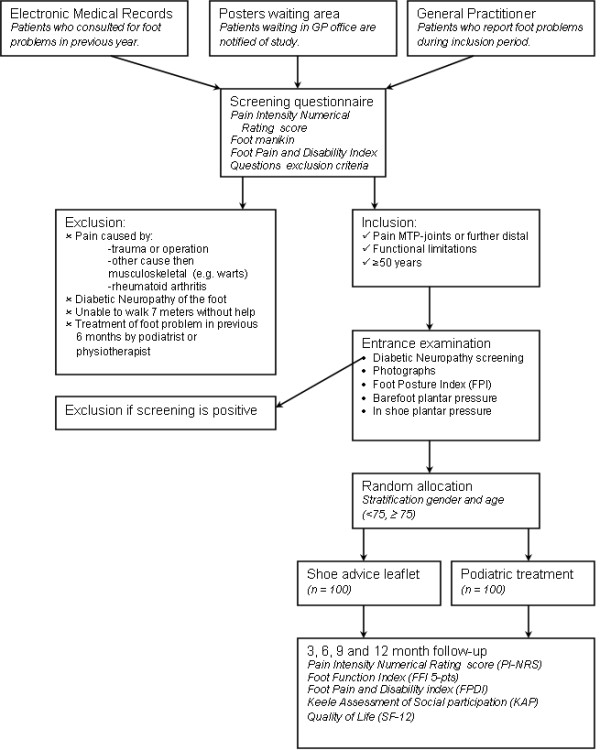
Design of the RCT

This study is designed as a pragmatic open randomised clinical trial with 12 months follow-up. Participants will be recruited via general practice clinics (figure 1); those who are eligible and give written consent to participation will be randomly assigned to either the intervention group or the control group. The Medical Ethics Committee of the VU University Medical Centre in Amsterdam has approved the design of this study (No. 2009/267).

### Participants

**Figure 2:**
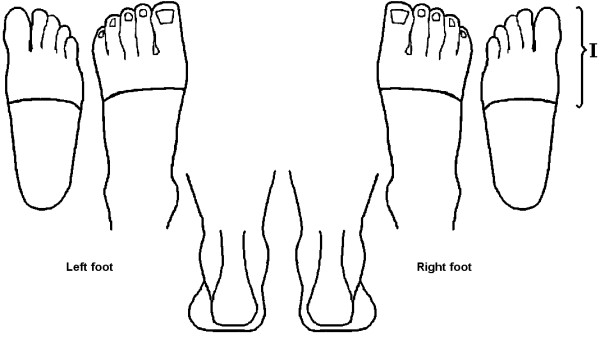
**Foot manikin screenings questionnaire **[5]. "I" is the inclusion area.

General practice clinics affiliated with the Academic Network for GPs of the VU University Medical Centre in Amsterdam will partake in this study. Recruitment of participants will be from patients who have visited their GP with non traumatic pain in the forefoot in the preceding year and those who will visit their GP during the recruitment period with a similar complaint. Participants, aged 50 or over, will have non-traumatic pain around the MTP-joints (figure 2) or further distally of at least one month's duration. The pain will be due to a musculoskeletal forefoot problem and the participants indicate to be functionally disabled because of this ailment. Participants will be excluded if they have received treatment for this problem in the previous six months or if the pain is caused by rheumatoid arthritis, a recent trauma, an operation, or by a non musculoskeletal problem (e.g. warts or a fungal infection of the foot). Additionally, patients with diabetic neuropathy of the feet or with foot problems that are deemed to be too serious by either the GP or by the study team to be treated in primary care will be excluded. Patients with rheumatoid arthritis or diabetic neuropathy are excluded from this trial because Dutch medical guidelines [16]indicate that these patients should be referred for podiatric care in all cases. Participants who are not able to walk 7 meters without a walking aid are also excluded, as they will not be able to perform the foot pressure measurements.

### Inclusion procedure

Potential participants will be recruited by three different methods. First, in all participating practices a retrospective search of the medical records will be carried out to identify all people aged 50 and over who have consulted their GP for a forefoot problem in the year preceding the start of the study. Second, all older people who consult their GP for forefoot problems during a period of 12 months following the start of the study will be considered for participation (prospective recruitment). Finally, all people visiting the general practice (for any reason) will be informed about the study by putting up posters in the waiting area of the GP clinics inviting patients to contact their GP if they have forefoot pain.

All potentially eligible patients will be invited to participate in the study and will receive comprehensive information about the study, a screening questionnaire and a pre-paid envelope to return the consent form and the questionnaire. Non-responders will receive a reminder after 2 weeks.

In addition to the assessment by the GP, inclusion and exclusion criteria will be assessed using self-report information from the screening questionnaire. Forefoot pain intensity is scored using a 0-10 point numerical rating scale and the location of pain is indicated on a foot manikin [17]. The area that will lead to inclusion in the study is shown in figure 2. Foot disability is assessed by the Foot Pain and Disability Index (FPDI) [17,18].

All patients who are potentially eligible for the study based on the screening questionnaire will be invited for a foot examination details of which will be discussed below (see Foot examination). The purpose of the foot examination is to measure baseline foot and pressure characteristics and to assess eligibility to participate in the trial. If the foot problem of the patient is considered to be too severe, and neither podiatric treatment nor shoe advice is considered adequate treatment, or if there are signs of any of the exclusion criteria like diabetic neuropathy, the patient will be referred back to their GP. If patients meet all eligibility criteria, written consent to participation in the trial will be obtained and a unique study number will be allocated to the participant.

### Foot examination

A foot examination will be performed at baseline before randomisation to assess eligibility. Additionally, 25 participants from the podiatry group will be asked to attend a second foot examination after three months to enable the process evaluation (see process evaluation of podiatric treatment). The presence of diabetic neuropathy in the forefoot will be ruled out based on tests using a 10 gram Weinstein monofilament and a 64 Hz tuning-fork. With the participant having his or her eyes closed, the skin on the plantar skin of the hallux and the MTP joints of I and V will be touched with the monofilament, and two out of three touches must be perceived. The vibrating tuning-fork is placed on the medial side of MTP1 and on the lateral side of MTP5; the vibration must be perceived for more than 5 seconds. Digital photographs of the ventral, lateral and medial side of the foot and shoes will be taken on a surface containing measurement lines in centimetres. Participants will be asked to bring the shoes they wear most often for this assessment and for the pressure measurements. Next, the Foot Posture Index will be assessed and scored according to Redmond et al.[19]. To assess pressure distribution patterns during barefoot and shod gait Emed-X (Novel gmbh, Münich, Germany) [20] and Pedar-X (Novel gmbh, Münich, Germany) [21,22] will be used. The Emed platform (4 sensors per cm2, sample frequency 100 Hz) is imbedded in a polyethylene walkway. A two-step protocol will be used for the barefoot pressure measurements, and each foot will be measured 3 times [23]. Each participant will be asked to walk at their preferred speed and with their preferred step length, in other words "as normally as possible". Everyone will have at least two practice runs per foot before the actual measurements are made. Participants are asked to look straight ahead to prevent targeting; if targeting is suspected, the measurement will be discarded and a new run will be executed. For the in-shoe pressure measurements an adequate size sensor-insole will be placed in the shoe (99 sensors/insole, 100 Hz). Participants will be asked to walk back and forth until twelve steps per foot are obtained. First steps, final steps and steps while turning will be excluded from the measurements.

### Podiatric treatment

The participants (n = 100; see Sample size) in the intervention group will be referred to a podiatrist to receive usual podiatric care. The treatment may consist of shoe advice, a silicone toe orthotic or corrective orthotics by means of an insole. Podiatrists are asked to follow the treatment protocol recommended by podiatric department of Fontys University of Applied Sciences. Each participant will be advised to contact the podiatrist if there are any problems with the treatment and an appointment will be made with each participant to examine progress and response to treatment after six to eight weeks. If needed, the treatment will be adjusted. The podiatrist will register details regarding the assessment of the forefoot problem, the diagnosis and treatment decisions on a standardised form.

### Control condition

Participants (n = 100; see Sample size) in the control group will receive a leaflet with shoe advice. The leaflet has been developed in cooperation with the GPs of the Academic Network of the VU University medical centre, the podiatric school of Fontys University of Applied Sciences and 8 podiatrists in the region. Shoe advice is part of the lifestyle advice that is frequently given by GPs to patients with foot problems [13]. Discussions with GPs prior to the trial indicated that this reflects a common minimal and first treatment option; general advice is given to wear well-fitting shoes of good quality and sometimes the patient's shoes are checked and commented on. Some GPs give more specific advice to adapt the shoe or to buy custom made shoe inlays or orthoses. In order to reflect usual care while ensuring optimal contrast between the two treatment groups, we will ask the GPs to refrain from specific individual advice. The participants will be advised to compare their shoes with recommended shoe wear presented in the leaflet and to purchase better fitting shoes if their shoes are not compatible with these recommendations. A reimbursement of € 25,- is given as an encouragement. All patients are invited to contact the research group or their GP for questions regarding the information leaflet. All participating GPs will receive a brief training session on how to provide information based on the leaflet and how to perform a shoe assessment.

### Primary and secondary outcome measures

The primary outcome measures will be forefoot pain intensity scored by the participant on an 11-point pain numerical rating scale (PI-NRS, where 0 = no pain and 10 = worst possible pain), foot function using the 5 point Foot Function Index (FFI-5pt) and foot disability using the FPDI [17,24,25]. The FFI-5pts has been translated into Dutch and validated for similar participants as an interview schedule. The FPDI has been validated for participants with foot problems but was not yet available in Dutch. Within the scope of this trial the FPDI has been translated into Dutch and will be validated according to methods proposed by Beaton et al.[26]. Secondary outcomes include social participation (Keele Assessment of Participation[27]) and Quality of Life (SF-12). Both primary and secondary outcome measures will be collected at baseline and after 3, 6, 9, and 12 months. Data on personal education and work history, shoe history and co-morbidity[28] are collected at baseline as possible effect modifiers or confounders.

### Process evaluation of podiatric treatment

Descriptive statistics will be used to describe the extent to which assessment of the forefoot problem and podiatric treatment decisions concur with the protocol, and if deviations (if any) can be explained.

For each patient in the podiatry treatment group, an expert panel of a podiatrist, a lecturer of the podiatry department of Fontys University for Applied Sciences and a human movement scientist will evaluate whether the diagnoses of the podiatrists are in accordance with the results of their assessment, and whether adequate decisions are made regarding treatment. For this evaluation, the notes of the podiatrists and the results of the entrance examination will be made available to the expert panel.

### Sample size

There are no evidence-based estimates for clinically important change in assessing forefoot pain or for clinically relevant differences between interventions for chronic foot pain. In a previous study it was estimated that a reduction of approximately two points on an 11-point pain intensity numerical rating scale represents a clinically important improvement [29]. We assume that the improvement in the podiatric treatment group will be one point larger than the improvement in the control group (a mean improvement of approximately 2 versus 1 point on the 0-11 point NRS, with an estimated standard deviation of 2.5). In this design the main question is "whether the podiatric treatment is more effective than the control treatment viz. a standardised shoe advice". Therefore we will perform a one-sided statistical test with the null hypothesis that podiatric treatment has either an equal outcome or a worse outcome as compared to standardised shoe advice, both outcomes having an equal clinical consequence [30].

In order to detect a 1 point difference in improvement between the groups after 12 months with a one-sided significance level of 0.05, and assuming a power of 0.8, an ICC between podiatrists and GPs of 0.05, a correlation of 0.50 between repeated measurements and a minimum of 5 patients per care provider, we would need complete data of 75 participants in each study group. We will enrol 2 × 100 patients to allow for a drop-out rate during follow-up of 25%. If every podiatrist will treat a minimum of 5 patients, 15 podiatrists will be needed to deliver the treatment in the entire intervention group.

In 17 practices of the Academic Network, with a total of n = 23,231 patients of 50 years or older, the diagnosis "foot problems" (ICPC 17) or free text words indicating foot problems were noted in the GP records of n = 497 patients within this age group. This incidence (21/1000) is in accordance with a previous estimate of 17/1000 [31]). We expect that about one third of all consulters presenting with foot problems have forefoot problems that meet our criteria [2,5], and assuming about 50% of these patients are eligible and willing to participate, a practice with a population of average age distribution can generate a minimum of 5 participants per year. This means that we will need to recruit at least 40 practices to participate in the trial to achieve the required number of patients.

### Treatment allocation and adherence

After providing informed consent, randomisation will be performed based on an allocation schedule that is generated before the start of the trial by a computerised random number generator using block randomisation with blocks of 8 or 4 with pre-stratification for gender and age (<75, ≥ 75). Since the age group ≥ 75 is expected to be smaller, a block of 4 has been chosen to increase the likelihood of equal distribution over the control and intervention groups. An independent research assistant will prepare coded sealed envelopes containing the treatment allocation. After baseline measurements the correct allocation envelope is opened by the participant. The foot examiner will be blinded for the random sequence and will not be informed of the block size ensuring concealed allocation of treatment. All GPs will be notified about the participants' allocated treatment, and will be asked to stimulate adherence to treatment whenever the participant contacts the GP during the intervention period. If symptoms do not improve despite adherence to treatment during the 3 months following randomisation, or with deterioration of the foot problem, the GP is free to provide another treatment or refer the participant for further treatment elsewhere.

### Data analysis

Multilevel analysis will be used to estimate the overall effect of podiatric treatment as compared to standardised shoe advice on the three primary outcome measures (PI-NTR, FFI-5pt and FPDI). Both clustering due to participants being treated by podiatrists and clustering due to repeated measurements within the same individuals will be taken into account. Results will be adjusted for differences in baseline similarity, if these occur. The effect of interest is the treatment × time interaction [32] where the primary focus will be on the primary outcome measures at 3 and 12 months' follow-up. Differences in secondary outcome will be estimated using similar statistical methods. All data will be analysed using an intention-to-treat approach. In all cases, a significance level of 5% is pre-stipulated.

## Discussion

In this paper we have described the design of a randomised clinical trial to compare the effects of two common treatments for forefoot pain in older people. During the design of this trial we had to make some decisions which could potentially influence the trial results. In order to explore the effectiveness of podiatric treatment on forefoot problems, it would be optimal to compare the podiatric treatment to a placebo treatment. However, we aim to enrol patients who consult their GP with a need for care of their forefoot problem. Consequently, including a no-treatment arm would not be an option in view of ethical reasons. GPs frequently only provide lifestyle advice for foot problems including the advice to wear well-fitting shoes [14]. By implementing a standardised minimal intervention strategy by means of a shoe leaflet we will reflect usual GP care for forefoot problems and not deny the participants a treatment for their forefoot problem. Participants allocated to this control treatment may be disappointed and we therefore decided to offer partial reimbursement of the costs of new shoes. We expect that this will reduce potential contamination between the control and intervention group and enhance treatment adherence in the control group. We will investigate treatment adherence and contamination in our process evaluation, perform a per protocol analysis as a secondary analysis.

It is conceivable that wearing better fitting shoes has a positive result on both foot function and foot pain. Nevertheless, in this study we are mainly interested to see if referral to podiatric treatment provides a better outcome than merely shoe advice. A problem we cannot resolve is that all subjects will be aware of how they are being treated, either by receiving standardised shoe advice or podiatric treatment. The GPs will be instructed to stimulate adherence to treatment whenever possible. If the foot pain of participants in either group does not respond to treatment after 3 months, the GPs are instructed to proceed with providing an alternative treatment. Possible changes of treatment are thoroughly documented.

Furthermore, although the podiatrists will be requested to adhere to treatment protocol, it is evident that podiatric treatment will be carried out by different therapists. In this design a maximum of 5 or 6 patients will be treated by a single podiatrist. This reduces the possible influence of a therapist effect on the outcome of the study, although the GPs would then need to refer to more podiatrists than they normally do. A multilevel analysis method will be used to estimate the 'therapist effect' (variation in treatment effect due to differences in podiatrists). Forefoot problems are very heterogeneous, and the various problems may respond differently to treatment. Although this will be analysed, the study sample will prove too small to provide conclusive evidence on any subgroup effects.

The strength of this study is that we created the design for a pragmatic trial which will compare two treatments that are most often advised by GPs: the advice to wear well-fitting shoes and podiatric treatment. Therefore, the results will contribute to clinical decision making by primary care professionals in patients with forefoot problems and it will provide information on the potential benefits of a referral for podiatric care.

## Competing interests

The authors declare that they have no competing interests.

## Authors' contributions

BvdZ will be responsible for data-collection and wrote, together with PE, the manuscript. DK has carried out the power analysis and helped to write the statistical paragraph. PE, KG, LP, DvdW and HvdH developed the original concept of the study and commented on the manuscript. The study design was further developed by BvdZ, PE, KG, LP, DvdW and HvdH. All authors have read and approved the final manuscript.
